# Exosomal circPTPRK promotes angiogenesis after radiofrequency ablation in hepatocellular carcinoma

**DOI:** 10.3389/ebm.2024.10084

**Published:** 2024-10-14

**Authors:** Yufeng Zhu, Qianru He, Ming Qi

**Affiliations:** ^1^ Department of General Surgery, The First Affiliated Hospital of Jinzhou Medical University, Jinzhou, Liaoning, China; ^2^ Liaoning Provincial Key Laboratory of Clinical Oncology Metabonomics, Jinzhou, Liaoning, China; ^3^ Zhuhai People’s Hospital Affiliated With Jinan University, Zhuhai, Guangdong, China; ^4^ Department of Ultrasound, The First Affiliated Hospital of Jinzhou Medical University, Jinzhou, Liaoning, China

**Keywords:** hepatocellular carcinoma, exosome, circPTPRK, radiofrequency ablation, angiogenesis

## Abstract

Radiofrequency ablation (RFA) is an effective treatment for hepatocellular carcinoma (HCC), but the recurrence rate remains high due to angiogenesis in residual cancer cells. We used thermal stimulation to simulate the post-RFA microenvironment. The expression profile of circRNAs between normal control HCC cell-derived exosomes and exosomes after heat stimulation were analyzed by RNA sequencing. Quantitative real-time PCR was applied to evaluate the expression of circPTPRK in exosomes and human umbilical vein endothelial cells (HUVECs). Then, the functions of heat-stimulated HCC cell-derived exosomes and exosomal circPTPRK on HUVECs were unveiled. Transcriptome sequencing was utilized to determine targeted genes of circPTPRK. Heat-stimulated HCC cell-derived exosomes augmented cell proliferation, migration, and angiogenesis of HUVECs. In total, 229 differentially expressed circRNAs were obtained, including 211 upregulated circRNAs and 18 downregulated circRNAs in heat-stimulated HCC cell-derived exosomes. The expression of circPTPRK was remarkably increased in heat-stimulated HCC cell-derived exosomes and the HUVECs incubated with them. Heat-stimulated HCC cell-derived exosomes with circPTPRK knockdown significantly inhibited cell proliferation, migration, and angiogenesis of HUVECs. Mechanistic studies indicated that PLA2G4E is a downstream target of circPTPRK, and PLA2G4E overexpression reversed the inhibitory effect of circPTPRK knockdown on HUVEC angiogenesis. Our results indicated that exosomal circPTPRK activated HUVEC angiogenesis by upregulating PLA2G4E expression.

## Impact statement

In this study, 229 differentially expressed circRNAs were obtained, including 211 upregulated circRNAs and 18 downregulated circRNAs in heat-stimulated HCC cell-derived exosomes. The expression of circPTPRK was remarkably increased in heat-stimulated HCC cell-derived exosomes and the HUVECs incubated with them. Heat-stimulated HCC cell-derived exosomes with circPTPRK knockdown significantly inhibited cell proliferation, migration, and angiogenesis of HUVECs. Mechanistic studies indicated that PLA2G4E is a downstream target of circPTPRK, and PLA2G4E overexpression reversed the inhibitory effect of circPTPRK knockdown on HUVEC angiogenesis. Our results indicated that exosomal circPTPRK activated HUVEC angiogenesis by upregulating PLA2G4E expression.

## Introduction

Hepatocellular carcinoma (HCC) is the most common primary liver cancer and the predominant cause of cancer death globally [1, 2]. At present, there are many treatments for HCC, but the prognosis remains relatively poor [3]. Furthermore, the incidence and mortality of HCC remain high. Radiofrequency ablation (RFA) has become one of the key approaches to prolonging the survival of HCC patients due to its minimally invasive, safe, and convenient use [4]. Studies have shown that thermal stimulation of RFA could promote angiogenesis of residual cancer. Therefore, the poor prognosis of some patients after ablation may be attributed to the abnormally vigorous angiogenesis under thermal stimulation, leading to the higher invasion of this part of the tumor [5]. Angiogenesis is the basic pathological feature of malignant tumors and performs a vital role in the development, metastasis, and occurrence of tumors [6]. Therefore, the elucidation of the mechanism of angiogenesis after RFA in HCC patients is of great value to improve the long-term efficacy of RFA.

Circular RNAs (circRNAs) are a type of noncoding RNA that are synthesized by “end-to-end” splicing of RNAs [7]. CircRNAs inhibit or promote cellular function by sponging microRNAs. Many studies have pointed out that circRNAs are closely associated with tumor progression. Guo et al. showed that circITCH (has-circ-0001141) acted as a repressor to inhibit HCC malignancy by sponging miR-184 [8]. Zhan et al. revealed that circRNA hsa_circRNA_103809 facilitated HCC development by modulating the miR-377-3p/FGFR1/ERK axis [9]. Moreover, it was shown that hsa_circRNA_100084 can be used as a miR-23a-5p sponge to stimulate IGF2 expression in HCC cells [10]. Notably, circRNAs can be identified in the blood and urine of patients, indicating that circRNAs may be diagnostic markers for human cancer [7]. However, limited studies have been conducted on circPTPRK, and its association with HCC remains unclear, meaning it requires further investigation.

Exosomes are extracellular vesicles with a diameter of 30–100 nm [11]. A large body of research has proven that exosomes are the communication bridge between cells and play an important role in the tumor microenvironment [12]. Exosomes participate in cancer-associated biological functions, such as cell proliferation, migration, and angiogenesis [13]. Exosomal circRNAs have been reported to play a crucial role in the development of numerous cancers, including gastric, colorectal, and pancreatic cancer [14]. For example, Exosomal circSTRBP derived from cancer cells promoted gastric cancer progression by modulating the miR-1294/miR-593-3p/E2F2 axis [15]. Exosomal circTUBGCP4 induced vascular endothelial cell tipping to foster angiogenesis and tumor metastasis through activation of the Akt signaling pathway [16]. Moreover, exosomal circRNAs are also closely related to the development of HCC. It has been shown that exosomal ANGPT2 secreted by HCC cells promoted tumor angiogenesis [17]. Evidence from recent studies has indicated that transfection of exosomal circRNA-100338 into recipient HUVECs could promote angiogenesis and subsequently affect HCC [18]. Adipocytes secreted exosomal circRNAs, which accelerated HCC tumor growth by repressing miR-34a and triggering the USP7/Cyclin A2 pathway [19]. Recently, exosomal lncRNA ASMTL-AS1 was reported to exacerbate the malignancy in residual HCC after insufficient RFA [20]. However, the effects of exosomal circRNA on angiogenesis after RFA remain obscure.

In the present study, we aimed to determine the role and regulatory mechanism of HCC cell-derived exosomal circRNAs in angiogenesis after RFA. We explored the role of MHCC-97H cell-derived exosomes after heat stimulation (HS-exo) in angiogenesis of human umbilical vein endothelial cells (HUVECs). RNA sequencing was carried out to determine differentially expressed circRNAs (DEcircRNAs) between normal control exosomes (NC-exo) and HS-exo. The function and mechanism of exosomal circPTPRK in HUVEC angiogenesis was determined via cell experiments and RNA sequencing. Our study may provide a hopeful therapeutic strategy for HCC recurrence after RFA.

## Materials and methods

### Cell culture and treatment

The human HCC cell lines (MHCC-97H and HepG2) and HUVECs were acquired from iCell Bioscience Inc. (Shanghai, China). MHCC-97H cells were cultivated in Dulbecco’s modified Eagle’s medium (DMEM) (CORNING, United States). HepG2 cells were cultivated in minimum Eagle’s medium (MEM) (CORNING, United States). The HUVECs were cultivated in endothelial cell medium (ScienCell, United States). MHCC-97H and HUVECs were appended with 10% fetal bovine serum (GIBCO, United States), 100 μg/mL streptomycin, and 100 U/mL penicillin in a 37°C incubator of 5% CO_2_.

To simulate the tumor microenvironment after RFA *in vitro*, MHCC-97H cells were cultivated at 42°C for 24 h (heat stimulation) according to a previous report [21], with some modification.

### Isolation of exosomes

Exosomes were extracted by the ultracentrifugation method in accordance with the previous report [18], with some changes. The collected medium was centrifuged at 300 g for 10 min at 4°C to remove the cell pellet. Afterwards, the supernatant was centrifuged at 2,000g for 10 min at 4°C and then 10,000 g for 10 min at 4°C to eliminate the dead cells and cell debris. Finally, the supernatant was centrifuged at 120,000 g for 2 h at 4°C. The precipitate was resuspended in pre-cooled 1×PBS and preserved at −80°C. The concentration of isolated exosomes was measured by the BCA kit (Beyotime, P0010S).

### Internalization of exosomes

MHCC-97H cells in the NC group and HS group were cultured at 37°C and 42°C for 24 h, respectively. Serum-free medium was replaced and supernatants were collected after 48 h culture to obtain exosomes via ultracentrifugation. Subsequently, the exosomes were supplemented with 1 μL of DiI fluorescent dye and incubated for 20 min at room temperature. HUVECs were incubated with exosomes labeled with DiI for 48 h. DAPI (Sangon) was used to stain cell nuclei. Finally, fluorescence microscope (NIKON, Japen) was employed to confirm the internalization of exosomes in HUVECS.

### RNA sequencing

TRIzol reagent was applied to separate total RNA from the collected NC-exo (20 μg/mL) and HS-exo (20 μg/mL). Construction of a cDNA library was conducted by utilizing TruSeq RNA Sample Prep Kit (Illumina, United States) and RNA sequencing was conducted on the Illumina HiSeq 2500 platform. Fast-QC was employed for sequencing raw data to evaluate the overall quality. Clean reads were mapped to GRCh38 with HISAT2 software. The differentially expressed circRNAs (DEcircRNAs) were acquired by utilizing DESeq2 with *P*-value <0.05 and |log_2_(Fold change)| > 1. Miranda/RNAhybird was applied to predict the target genes of DEcircRNAs. Gene ontology (GO) and Kyoto encyclopedia of genes and genomes (KEGG) enrichment analysis was used to analyze the function and enrichment pathways of target genes of DEcircRNAs. To determine the downstream targets of circPTPRK, RNA sequencing was performed with si-NC and si-circPTPRK HUVECs (n = 3). The differentially expressed genes were acquired by utilizing DESeq2 with *P*-value <0.05 and |log_2_(Fold change)| > 1. GO and KEGG enrichment analysis was employed to analyze the function and enrichment pathways of target genes of circPTPRK.

### Cell transfection

Small interfering (si) RNAs targeting circPTPRK (circPTPRK siRNA-1/2/3), siRNA negative control (si-NC), and PLA2G4E overexpression plasmid were acquired from Shanghai GenePharma Co., Ltd. (China). Cells were placed in 6-well plates with 30 × 10^4^ cells each well. Transfection was executed by employing Lipofectamine 2000 (Invitrogen, United States) in line with the manufacturer’s protocol.

### Quantitative real-time PCR (qRT-PCR)

Total RNA was segregated with TRIzol reagent (Invitrogen). Reverse transcription reaction was conducted using Thermo Scientific RevertAid First Strand cDNA Synthesis Kit (Thermo). The qRT-PCR reaction was conducted with the SYBR Green PCR kit (Roche) on the Applied Biosystems Q6 real-time PCR system (United States). The reaction conditions consisted of 95°C for 10 min, followed by 40 cycles of 95°C for 15 s, and 60°C for 60 s. GAPDH was regarded as an internal control. The relative expression of circRNA and genes was detected using the 2^−ΔΔCT^ method. The primers are listed in Supplementary Table S1.

### Cell counting kit-8 (CCK-8) assay

Cell proliferation was evaluated by CCK-8. HUVEC were placed in 6-well plates and co-cultured with exosomes (20 μg/mL) for 48 h. Afterward, 10 μL of CCK8 (Beyotime, China) solution was appended to each well. After 2 h of incubation at 37°C, the absorbance was read out with a microplate spectrophotometer (Bio-Rad, United States) at 450 nm.

### Transwell assay

Transwell migration assays were conducted in 24-well plates (Corning, United States). HUVECs and exosomes (20 μg/mL) were plated on the upper chambers. Endothelial cell medium comprising 10% FBS was appended to the lower chambers. After incubation for 48 h, cells were fixed with 4% paraformaldehyde, and subsequently dyed with crystal violet solution. In the end, the cells were photographed and counted using a microscope.

### Wound scratch assay

The transfected cells were seeded in 6-well plates, reaching a cell density exceeding 90%. After 24 h, scratches were drawn on the cells with a pipette tip, followed by washing the cells twice with PBS. Subsequently, images were captured at 0 and 24 h using a Nikon microscope.

### Tube formation assay

The impact of HS-exo and exosomal circPTPRK on angiogenesis was examined by the tube formation assay. HUVECs were incubated in serum-free medium for 12 h and subsequently transferred to 48-well plates precoated with matrigel (356230, Corning) for 10 h. Exosomes (20 μg/mL) from different treatments were added into HUVEC and incubated for 48 h. The formation of blood vessels was observed by utilizing a microscope, and the vascular branches were analyzed by utilizing ImageJ software.

### Statistical analysis

Statistical analysis was conducted by utilizing GraphPad Prism 9. Data were displayed as mean ± standard deviation (SD). The differences between the two groups were compared by student t-test, and the three groups were determined by the one-way ANOVA. A *P*-value less than 0.05 was considered as statistically significant.

## Results

### HCC cell-derived exosomes produced after heat stimulation promote HUVEC proliferation, migration, and tube formation

**FIGURE 1:**
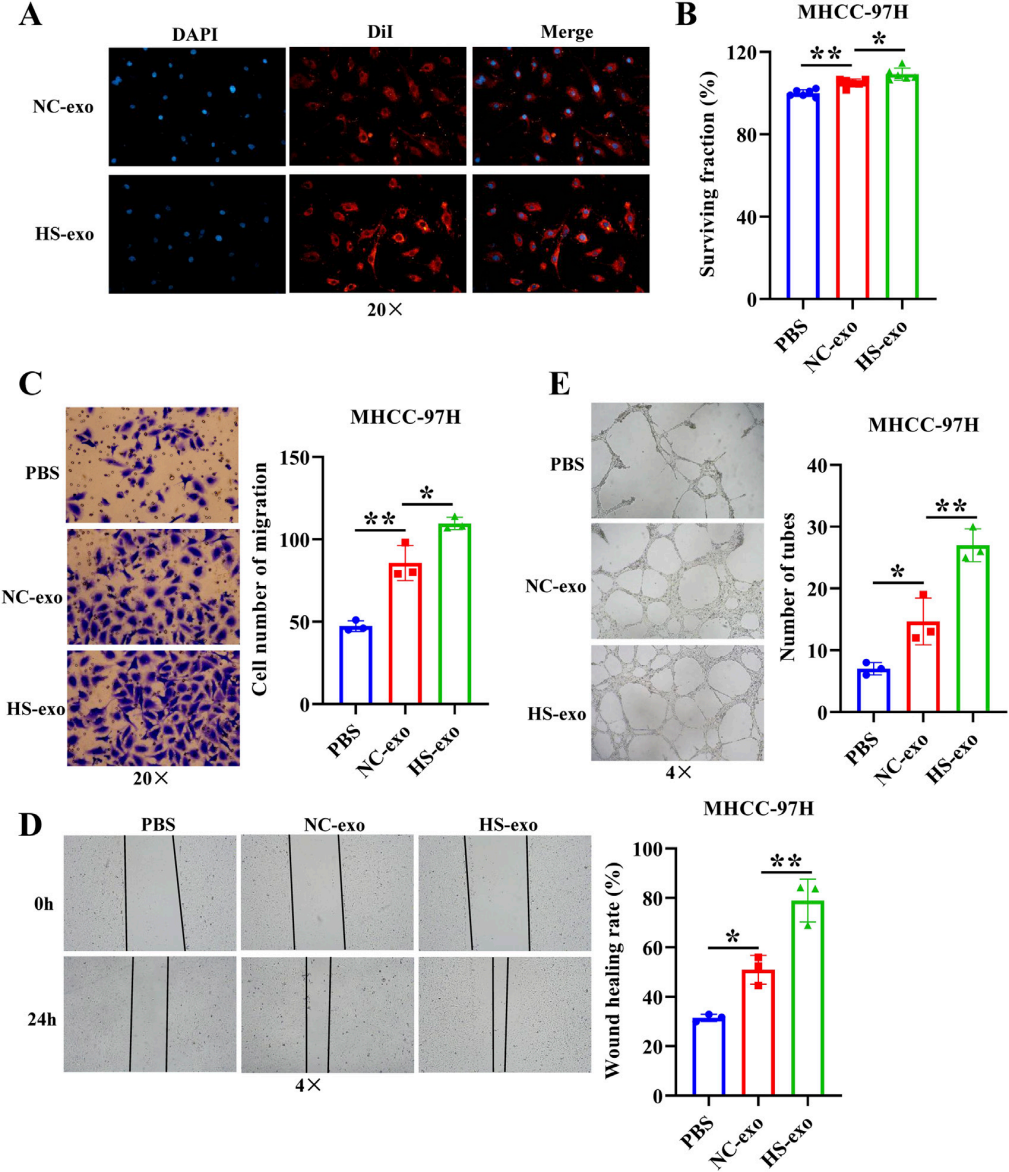
Effects of exosomes produced after heat stimulation on HUVEC function. **(A)** Exosome absorption by HUVECs was observed by fluorescence microscope. n = 3. **(B)** The effect of exosomes produced after thermal stimulation on HUVEC proliferation was detected by CCK-8. n = 6. Transwell assay **(C)** and wound scratch assay **(D)** were applied to examine the effect of exosomes produced after thermal stimulation on HUVEC migration. n = 3. **(E)** Tube formation assay was employed to examine the impact of exosomes produced after thermal stimulation on tube formation ability of HUVECs. n = 3. NC-exo: Normal control exosomes derived from human hepatocellular carcinoma cell line MHCC-97H. HS-exo: Exosomes produced after thermal stimulation. *p < 0.05; **p < 0.01.

In order to determine whether HS-exo regulate endothelial cell function, we first performed exosome tracer experiments. The results displayed that HS-exo were absorbed by endothelial cells (Figure 1A). Then, we used CCK8, transwell, wound scratch assay, and tube formation cell function experiments to examine their effects on HUVEC proliferation, migration, and tube formation capacity. The results showed that HS-exo dramatically boosted HUVEC proliferation (1.04-fold), migration (1.28-fold in transwell assay, and 1.55-fold in wound scratch assay), and tube formation capacity (1.84-fold) relative to NC-exo (Figures 1B–E). Together, HS-exo promoted endothelial cell proliferation, migration, and tube formation.

### Analysis of differentially expressed circRNAs in exosomes after heat stimulation

**FIGURE 2:**
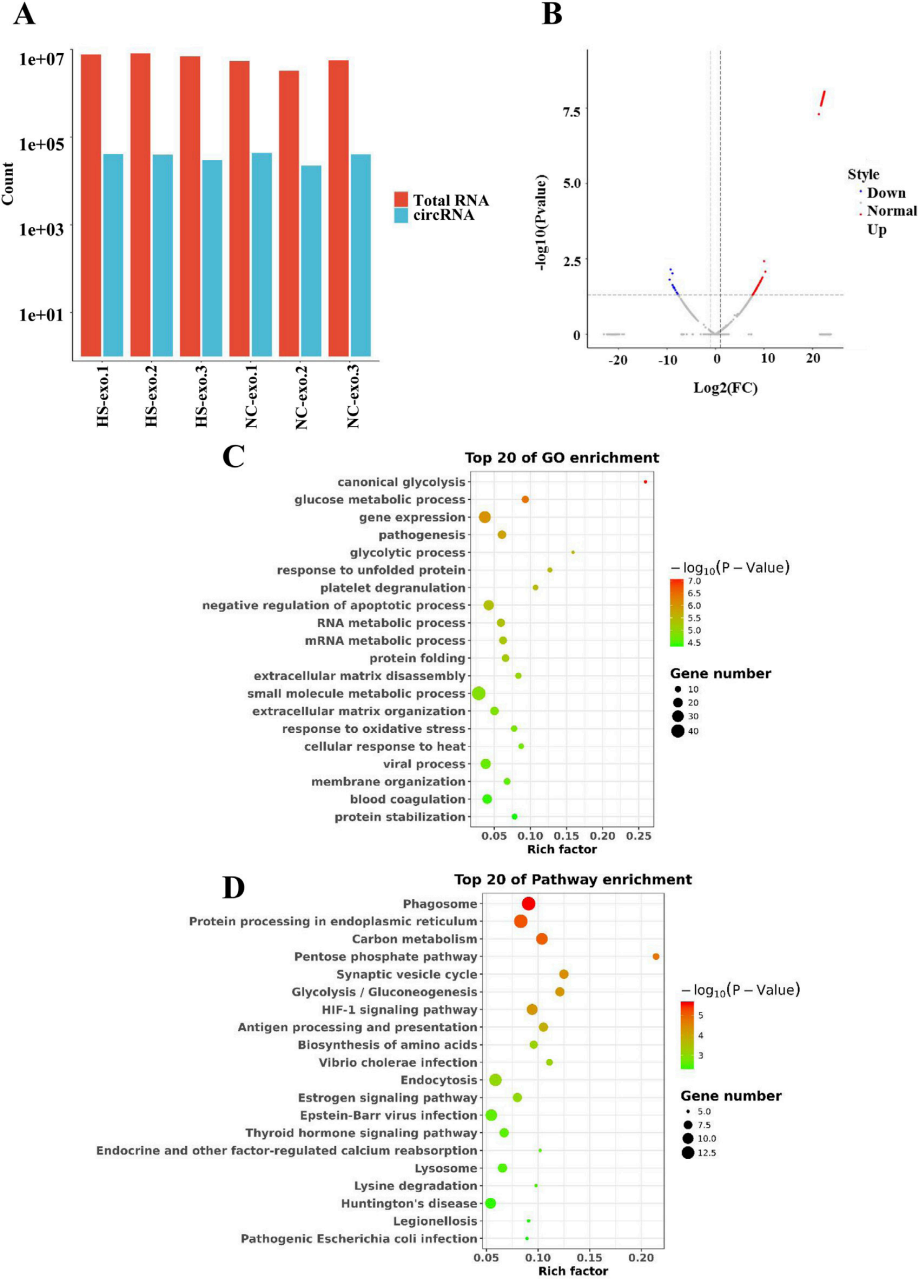
Analysis of differentially expressed circRNAs (DEcircRNAs) in exosomes after heat stimulation. **(A)** The comparison of total RNA vs. cirRNA in NC-exo and HS-exo. **(B)** Volcano plot of DEcircRNAs between NC-exo and HS-exo (n = 3). **(C)** GO analysis of target genes of DEcircRNAs. **(D)** KEGG analysis of target genes of DEcircRNAs.

We conducted RNA sequencing of normal control HCC cell-derived exosomes and exosomes produced after thermal stimulation. The comparison of total RNA vs. cirRNA in NC-exo and HS-exo is shown in Figure 2A. In comparison with the normal control exosomes, 229 circRNAs were differentially expressed in the exosomes produced after heat stimulation. Among them, 211 circRNAs were strikingly up-regulated, whereas 18 were obviously down-regulated in exosomes produced after thermal stimulation (Figure 2B). GO analysis revealed that target genes of DEcircRNAs were principally implicated in the mRNA metabolic process, cellular response to heat, and gene expression (Figure 2C). KEGG enrichment analysis unveiled that target genes of DEcircRNAs were primarily enriched in Protein processing in endoplasmic reticulum, Pentose phosphate pathway, and HIF−1 signaling pathways (Figure 2D).

### circPTPRK was up-regulated in exosomes after heat stimulation

**FIGURE 3:**
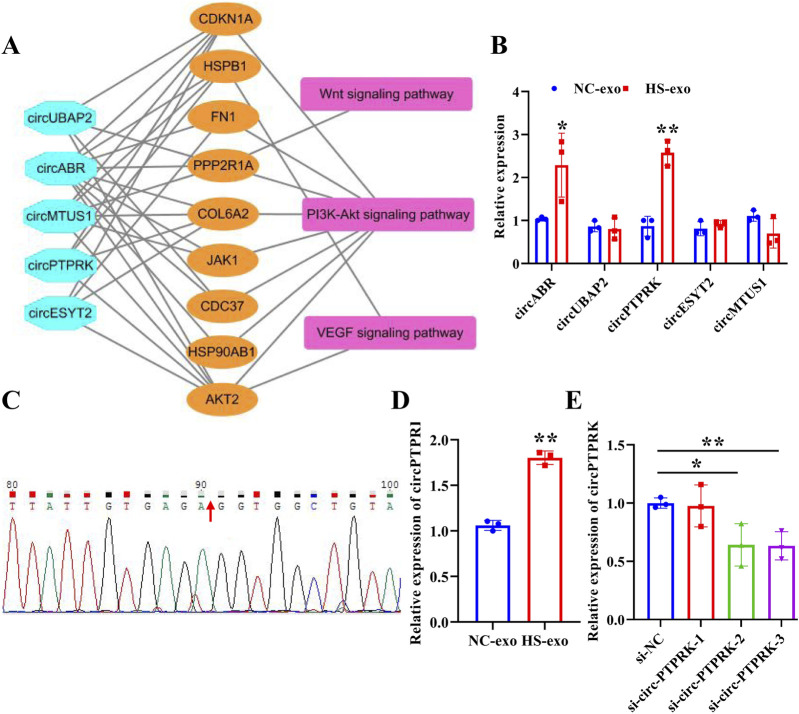
circPTPRK expression was up-regulated in exosomes after heat stimulation. **(A)** The network diagram of circRNAs-mRNAs-pathways. **(B)** qRT-PCR was employed to examine the expression of candidate circRNAs in NC-exo and HS-exo. n = 3. **(C)** Sanger sequencing confirmed the circular structure of circPTPRK. **(D)** Expression of circPTPRK was tested by qRT-PCR in HUVECs incubated with exosomes produced after heat stimulation. n = 3. **(E)** The interference efficiency of circPTPRK was confirmed in MHCC-97H cells by qRT-PCR. n = 3. *p < 0.05; **p < 0.01.

To determine the pivotal circRNAs that modulate angiogenesis, a “circRNAs—target genes—angiogenesis-related pathways” network was constructed (Figure 3A). Five circRNAs (circABR, circUBAP2, circPTPRK, circESYT2, and circMTUS1) were picked for qRT-PCR validation. The results showed that circPTPRK was significantly upregulated in HS-exo with 2.58-fold change, showing the largest fold difference (Figure 3B). Therefore, circPTPRK was selected for subsequent experiments. Sanger sequencing verified the circular structure of circPTPRK (Figure 3C). circPTPRK expression was remarkably enhanced in HUVECs incubated by HS-exo (Figure 3D). In order to explore whether exosomal circPTPRK can regulate the function of endothelial cells, circPTPRK siRNA-1/2/3 and si-NC were transfected into MHCC-97H cells. qRT-PCR results uncovered that the expression of circPTPRK was dramatically down-regulated in MHCC-97H cells transfected with circPTPRK siRNA-3 with the largest difference (Figure 3E). Therefore, circPTPRK siRNA-3 was selected to knockdown circPTPRK.

### Exosomal circPTPRK promotes HUVEC proliferation, migration, and tube formation

**FIGURE 4:**
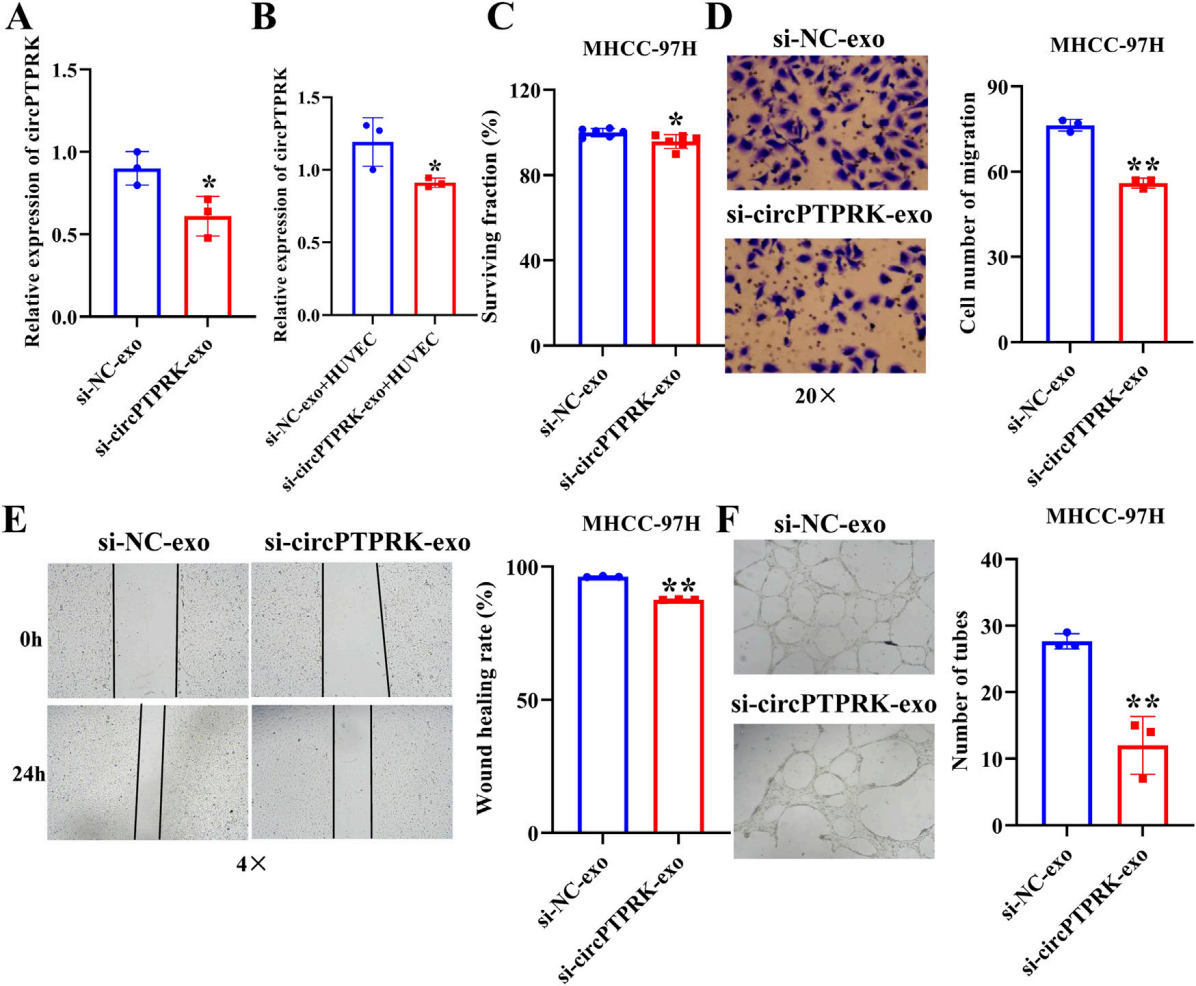
Heat-stimulated MHCC-97H cell-derived exosomes regulate HUVEC function through circPTPRK. **(A)** The expression of circPTPRK in circPTPRK knockdown exosomes was assessed via qRT-PCR. n = 3. **(B)** circPTPRK expression was tested by qRT-PCR in circPTPRK knockdown exosome-incubated HUVECs. n = 3. **(C)** The impact of circPTPRK on cell proliferation of HUVECs was evaluated via CCK-8. n = 6. The effect of circPTPRK on cell migration of HUVECs was detected by transwell assay **(D)** and wound scratch assay **(E)**. n = 3. **(F)** Tube formation assay was utilized to evaluate the impact of circPTPRK on tubular formation ability of HUVECs. n = 3. *p < 0.05; **p < 0.01.

**FIGURE 5:**
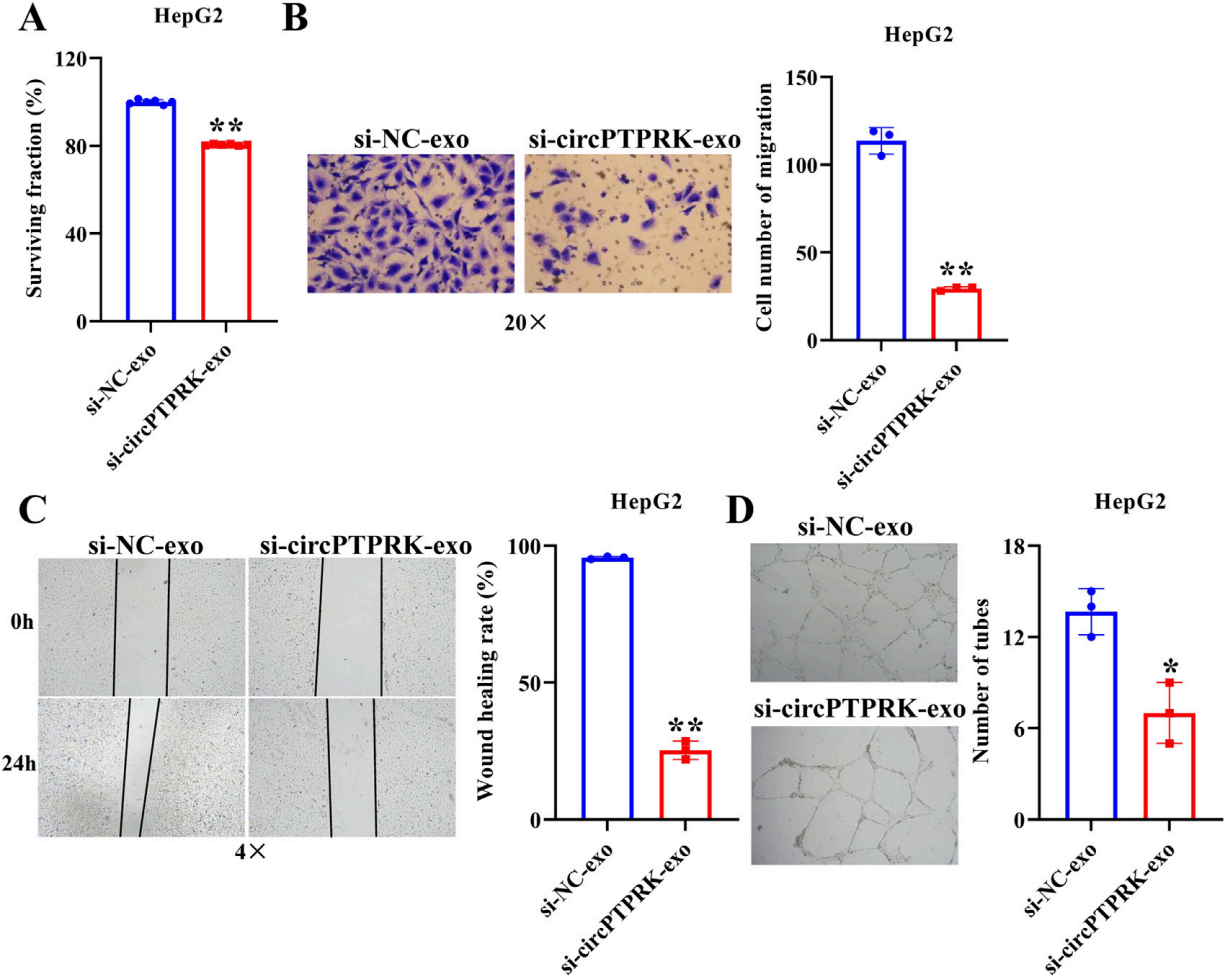
HepG2 cell derived-exosomal circPTPRK promotes HUVEC angiogenesis. **(A)** The impact of circPTPRK on proliferation of HUVECs was evaluated via CCK-8. n = 6. The effect of circPTPRK on the migration of HUVECs was detected by transwell assay **(B)** and wound scratch assay **(C)**. n = 3. **(D)** Tube formation assay was utilized to evaluate the impact of circPTPRK on tubular formation ability of HUVECs. n = 3. *p < 0.05; **p < 0.01.

We further investigated whether exosomes produced after heat stimulation function on endothelial cells by carrying circPTPRK. qRT-PCR results verified that circPTPRK expression was markedly diminished in circPTPRK knockdown exosomes relative to the normal control group (Figure 4A). circPTPRK expression was also prominently diminished in HUVECs incubated with circPTPRK knockdown exosomes compared with control exosome-incubated HUVECs (Figure 4B). Next, we used CCK8, transwell, wound scratch assay, and tube formation cell function experiments to examine the role of two cell line (MHCC-97H and HepG2)-derived exosomal circPTPRKs in HUVEC proliferation, migration, and tube formation. Results showed that MHCC-97H cell-derived exosomes with circPTPRK knockdown remarkably reduced HUVEC proliferation, migration, and tube formation relative to the control exosomes (Figures 4C–F). The same results were observed in HUVECs after HepG2 cell-derived exosomes with circPTPRK knockdown treatment (Figures 5A–D). In conclusion, exosomes produced by heat stimulation augmented HUVEC proliferation, migration, and tube formation through circPTPRK.

### Analysis of circPTPRK target genes

Transcriptome sequencing was carried out on circPTPRK knockdown and control HUVECs. In contrast with the control group, 305 target genes were differentially expressed in circPTPRK knockdown HUVECs. Among them, 195 target genes were up-regulated and 110 target genes were down-regulated after circPTPRK knockdown (Supplementary Figures S1A, B). To discover the biological function of differentially expressed target genes, we conducted functional enrichment and pathway analysis of target genes. GO analysis showed that these differentially expressed target genes were chiefly implicated in hormone biosynthesis and phosphorylation regulation (Supplementary Figure S1C). KEGG enrichment analysis displayed that differentially expressed target genes were principally enriched in T cell receptor, VEGF, and TGF-beta signaling pathways (Supplementary Figure S1D).

### Overexpression of PLA2G4E reverses the effect of exosomal circPTPRK knockdown on proliferation, migration, and tube formation of HUVECs

**FIGURE 6:**
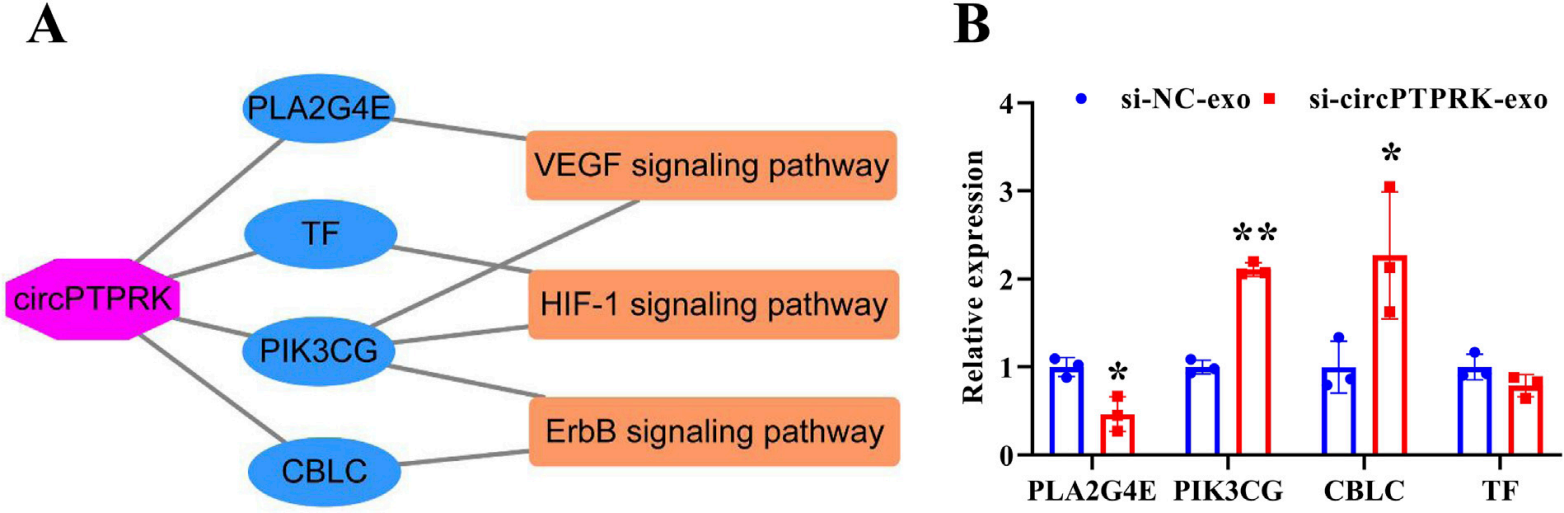
Screening of circPTPRK target genes. **(A)** The network of circPTPRK-target gene-angiogenesis-related signaling pathway. **(B)** qRT-PCR was applied to test the expression of candidate target genes in circPTPRK knockdown HUVECs. n = 3, *P < 0.05; **P < 0.01.

**FIGURE 7:**
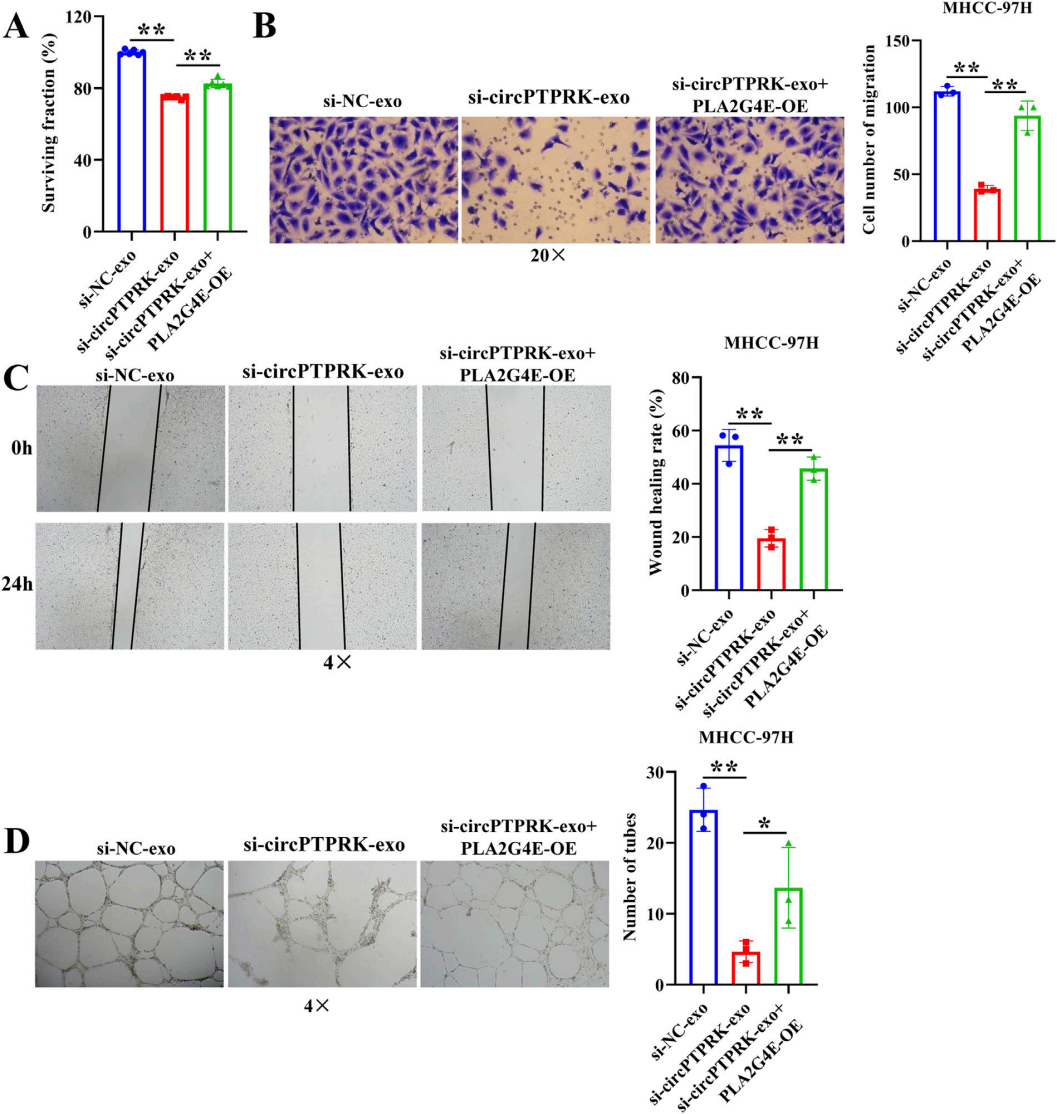
MHCC-97H cell-derived exosomal circPTPRK promotes HUVEC angiogenesis by modulating PLA2G4E expression. **(A)** The impact of exosomal circPTPRK knockdown and PLA2G4E overexpression on cell proliferation of HUVECs was evaluated via CCK-8. n = 6. The effect of exosomal circPTPRK knockdown and PLA2G4E overexpression on cell migration of HUVECs was detected by transwell assay **(B)** and wound scratch assay **(C)**. n = 3. **(D)** Tube formation assay was utilized to evaluate the impact of exosomal circPTPRK knockdown and PLA2G4E overexpression on tubular formation ability of HUVECs. n = 3. *p < 0.05; **p < 0.01.

To determine the regulatory mechanism of circPTPRK in HUVEC angiogenesis, we screened target genes enriched in angiogenesis-related pathways through RNA sequencing data (Figure 6A) and selected four target genes with large differences and high expression levels for qRT-PCR validation. The results revealed that the expression of PLA2G4E and CBLC was in line with the transcriptome sequencing results. circPTPRK knockdown significantly decreased PLA2G4E expression, while it increased CBLC expression in HUVECs (Figure 6B). PLA2G4E was selected for subsequent studies. To further investigate whether MHCC-97H cell-derived exosomal circPTPRK regulates HUVEC angiogenesis by targeting PLA2G4E, we performed rescue experiments. The results showed that exosomal circPTPRK knockdown remarkably repressed proliferation, migration, and tube formation of HUVECs, which was partly reversed by PLA2G4E overexpression (Figures 7A–D). Overall, exosomal circPTPRK knockdown inhibited HUVEC angiogenesis by regulating PLA2G4E expression.

## Discussion

**FIGURE 8:**
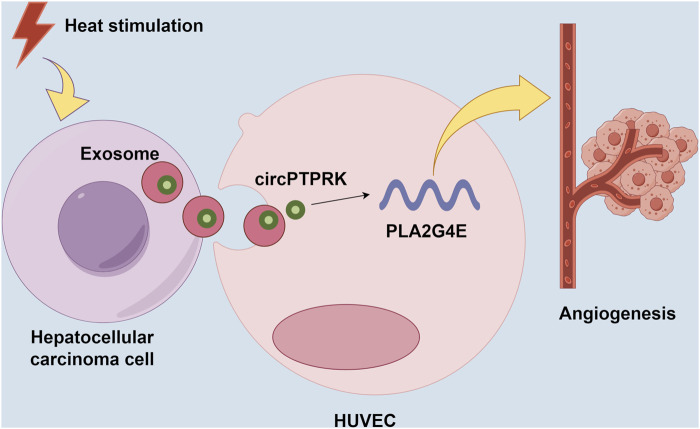
Schematic model for the mechanism by which exosomal circPTPRK promotes angiogenesis after radiofrequency ablation in hepatocellular carcinoma.

HCC is a common tumor worldwide, with high morbidity and mortality [22]. In this study, we discovered that HCC cell-derived exosomes produced after thermal stimulation promoted cell proliferation, migration, and tube formation of HUVECs. Moreover, circPTPRK was markedly upregulated in the HS-exo group relative to the NC-exo group. Exosomal circPTPRK knockdown could significantly suppress proliferation, migration, and tube formation of HUVECs. Mechanistically, circPTPRK targeted PLA2G4 and exosomal circPTPRK knockdown repressed HUVEC angiogenesis by regulating PLA2G4E expression (Figure 8). Therefore, we believe that exosomal circPTPRK could be a potential therapeutic target for HCC.

Angiogenesis is defined as a marker in HCC progression and can be used as a therapeutic target [23]. In our study, we discovered that exosomal circPTPRK could be transported to HUVECs and play its role in promoting angiogenesis. Evidence has shown that exosomal circRNAs can regulate angiogenesis by enhancing the communication between HCC cells and HUVECs [18]. Additionally, exosomes regulated cell-to-cell communication, changed the tumor microenvironment, and stimulated angiogenesis through proteins, nucleic acids, and other substances [24]. Similarly, anti-angiogenesis has shown great potential in the treatment of other malignancies. The results of Yu et al. displayed that exosomal circATP10A was a biomarker for angiogenesis in multiple myelomas [25]. Previous research indicated that circCCAC1 participated in cholangiocarcinoma progression, induced angiogenesis, and damaged vascular endothelial barriers [26]. Li et al. pointed out that gastric-cancer-derived exosomal circ29 promoted angiogenesis by modulating the miR-29a/VEGF axis in endothelial cells [27]. Given the vital role of angiogenesis in tumor progression and the negative impact of existing anti-angiogenic drugs, there is an urgent need to continuously search for new anti-angiogenic targets in the future.

In this study, we discovered that target genes of circPTPRK were mainly involved in VEGF and TGF-beta signaling pathways. VEGF is a crucial modulator of angiogenesis and exerts a vital role in angiogenesis [28]. A recent study reported that PinX1 inhibited renal cancer angiogenesis via the VEGF signaling pathway [29]. Another study found that TRPV3 facilitated angiogenesis via the HIF-1α-VEGF signaling pathway in non-small-cell lung cancer [30]. The TGF-beta pathway is also vital to tumor angiogenesis. Chen et al. found that Prrx1 contributed to stemness and angiogenesis via the TGF-β/smad pathway in glioma [31]. Therefore, we presumed that exosomal circPTPRK promoted angiogenesis via VEGF and TGF-beta signaling pathways in HCC.

CircRNAs primarily function in cancers via targeting miRNAs to increase the expression of mRNAs. For example, circCRIM1 contributed to the development of esophageal squamous cell carcinoma by upregulating TCF12 [32]. Silencing of circCCNB1 repressed cervical cancer development by increasing SOX4 expression [33]. circABCC4 accelerated the malignant behavior of prostate cancer by sponging miR-1182 to upregulate FOXP4 expression [34]. In our study, we found that exosomal circPTPRK knockdown inhibited HUVEC angiogenesis by downregulating PLA2G4E expression.

Certainly, there were a few limitations in our study. First, the effect of exosomal circPTPRK *in vivo* remains unknown. Second, the molecular mechanism of circPTPRK in angiogenesis should be studied further.

In conclusion, our study suggested that HS-exo participated in the modulation of HUVEC proliferation, migration, and angiogenesis by transmitting circPTPRK. Consequently, circPTPRK is considered a potential therapeutic target for HCC treatment after RFA.

## Data Availability

All the data obtained and materials analyzed in this research are available from the corresponding author upon reasonable request.

## Supplementary material

SUPPLEMENTARY FIGURE S1 Expression and functional analysis of circPTPRK target genes. **(A)** Volcano plot of circPTPRK-targeted genes. Transcriptome sequencing was performed on circPTPRK knockdown and control HUVECs (n = 3). **(B)** Cluster map of circPTPRK-targeted genes. **(C)** GO analysis of differentially expressed circPTPRK target genes. **(D)** KEGG analysis of differentially expressed circPTPRK target genes.
