# Supplementary material for: Exosomal circPTPRK promotes angiogenesis after radiofrequency ablation in hepatocellular carcinoma
Source: Exp Biol Med (Maywood). 2024 Oct 14;249:10084. doi: 10.3389/ebm.2024.10084 (PMC11514274; doi:10.3389/ebm.2024.10084)
Supplement: Supplementary file 1 [file DataSheet2.PDF]

Table S1 Primer sequences for qRT-PCR.

Primer	Sequences (5'to3')
GAPDH-F	AGAAGGCTGGGGCTCATT
GAPDH-R	AGAAGGCTGGGGCTCATT
circUBAP2-F	GCAACACAACAATCAGATGGC
circUBAP2-R	TCTTCCAAGCCCCTTTGAGT
circABR-F	TTTGAGATCGAGCTGGAGGG
circABR-R	AAGAACCCCGAGAGAACCAG
circPTPRK-F	GTTTGCCGCTTCCTTCAGAT
circPTPRK-R	GCCCCTGGACCATCATCAAA
circESYT2-F	TGACAAAGACCAAGCCAACG
circESYT2-R	ACTTTCCCTTGACAAGTCCCT
circMTUS1-F	TGAGGCGGAACAGTGACAAT
circMTUS1-R	ATGAGAGGGTGGGCAAAATG
PLA2G4E-F	GCCAGACAGACTGTTTTGTGA
PLA2G4E-R	ACTCAACTCTAGCACGTTCTTCA
PIK3CG-F	GGCGAAACGCCCATCAAAAA
PIK3CG-R	GACTCCCGTGCAGTCATCC
CBLC-F	CCACACCTTCTGGAGGGAAAG
CBLC-R	GTCGAACTCGAAGATGGACAC
TF-F	GGTGGCAGAGTTCTATGGGTC
TF-R	ACAGTAAAGTAAGCCTATGGGGA
