# Supplementary figures and images for: Exosomal circPTPRK promotes angiogenesis after radiofrequency ablation in hepatocellular carcinoma
Source: Exp Biol Med (Maywood). 2024 Oct 14;249:10084. doi: 10.3389/ebm.2024.10084 (PMC11514274; doi:10.3389/ebm.2024.10084)

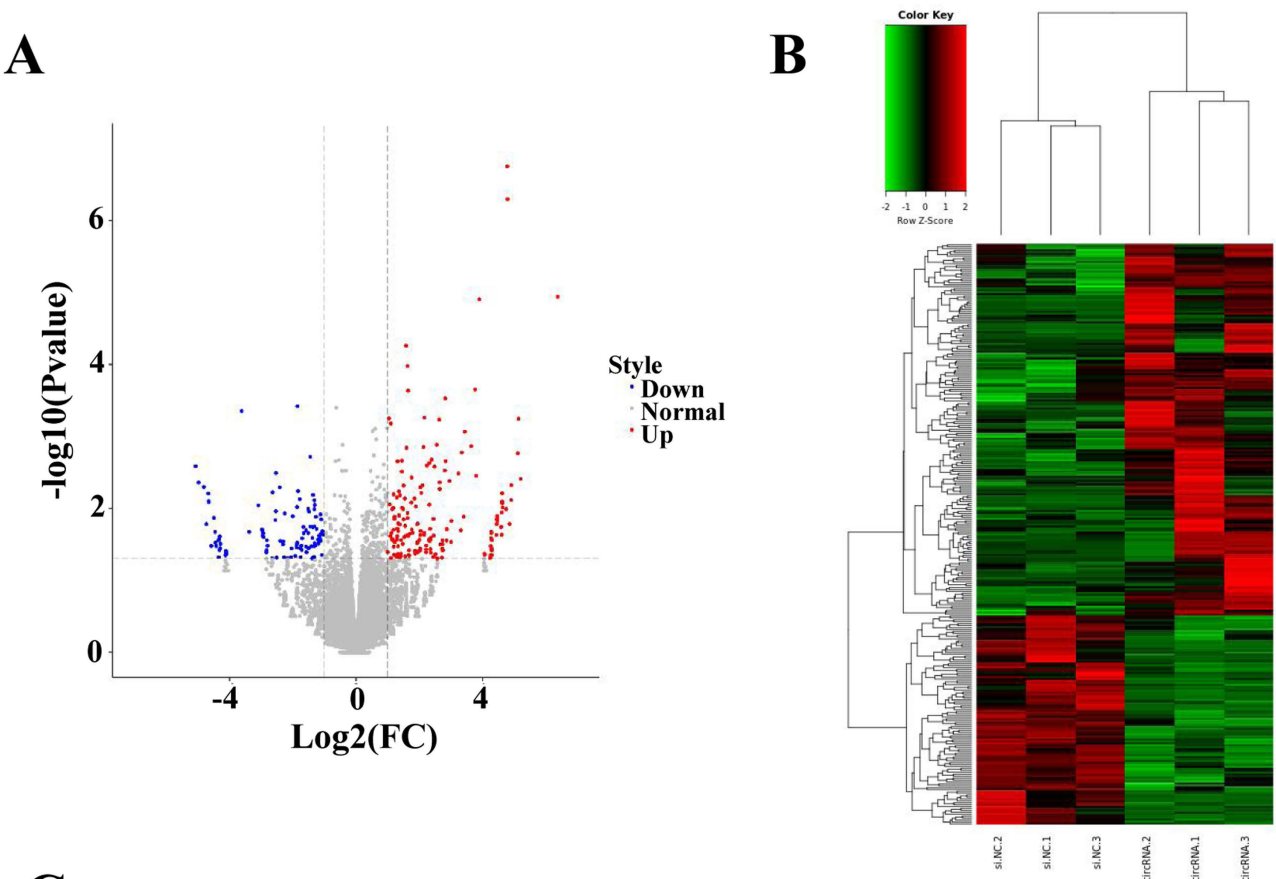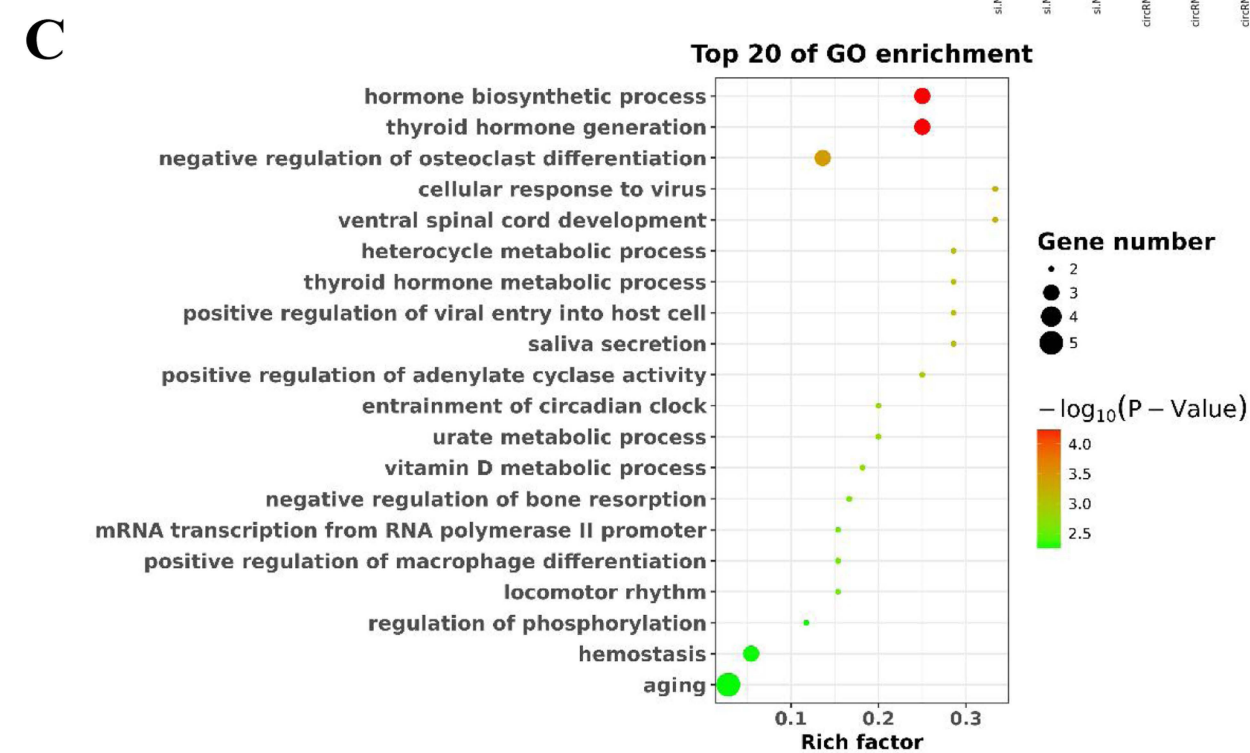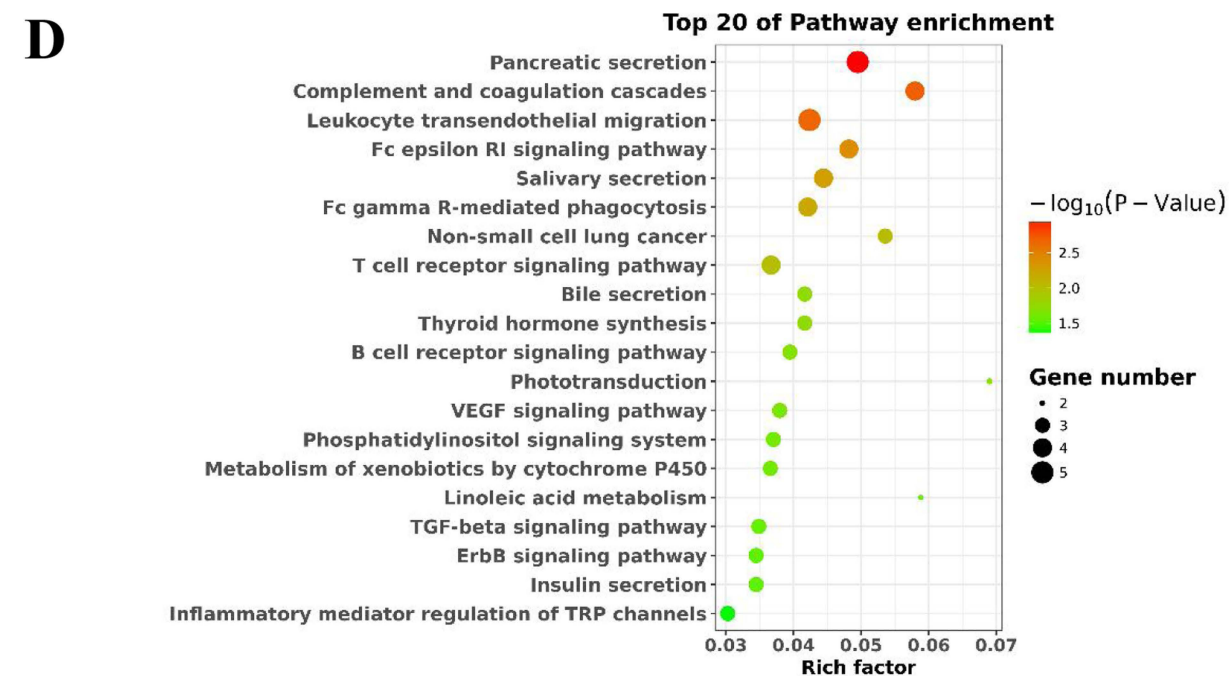

Supplement: Supplementary file 2 [file DataSheet1.PDF]
